# Short QT syndrome: The current evidences of diagnosis and management

**DOI:** 10.1002/joa3.12439

**Published:** 2020-10-06

**Authors:** Ivana P. Dewi, Budi B. Dharmadjati

**Affiliations:** ^1^ Faculty of Medicine Universitas Airlangga Surabaya Indonesia; ^2^ Faculty of Medicine Duta Wacana Christian University Yogyakarta Indonesia; ^3^ Department of Cardiology and Vascular Medicine Dr. Soetomo General Hospital Surabaya Indonesia

**Keywords:** channelopathies, short QT syndrome, sudden cardiac death

## Abstract

There are many cardiac arrhythmias and sudden cardiac death (SCD) related to channelopathies or ion channel disorders. Short QT syndrome (SQTS) is an inherited cardiac channelopathy principally caused by defective functioning of both potassium–calcium ion channel that lead to abnormal shortening of QT interval, and an increased risk of ventricular and atrial arrhythmias. Tall T waves in all lead electrocardiogram (ECG), peaked T waves, and narrow‐based T waves that are reminiscent of the typical “desert tent” T waves of hyperkalemia are frequently associated with SQTS. Diagnosis is based on patient's family history, evaluation of symptoms (palpitations and cardiac arrest), and 12‐lead ECG. It can be time challenging because of the wide range of QT interval in healthy subjects. Implantable cardioverter defibrillator (ICD) is the first‐line therapy in SQTS. Quinidine has the potential to be an effective pharmacological therapy for SQTS patients, especially in young children who are not feasible in ICD implantation, because of the ability to prolong QT interval.

## Graphical abstract

Short QT syndrome (SQTS) is an inherited cardiac channelopathy principally caused by defective functioning both potassium–calcium ion channel that lead to abnormally short QT interval, and an increased risk of atrial and ventricular arrhythmias. Diagnosis is based on the evaluation of symptoms (cardiac arrest and palpitations), patient's family history, and 12‐lead ECG and can at times be challenging due to the wide range of QT interval in healthy subjects. ICD is the first‐line therapy in SQTS. The ability of quinidine to prolong the QT interval has the potential to be an effective pharmacological therapy for short QT patients.

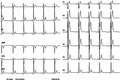

## 1 INTRODUCTION

Congenital arrhythmogenic abnormalities are a frequent cause of sudden cardiac death (SCD). This disorder is related to healthy and young people. It has been considered as one of the most common causes of sudden death in young athletes.^1^ These disorders include, Brugada syndrome, long QT syndrome (LQTS), catecholaminergic polymorphic ventricular tachycardia, and short QT syndrome (SQTS).^2^

SQTS is a cardiac channelopathy disorder characterized by short QT intervals and an increased risk of life‐threatening arrhythmias. Although often underdiagnosed, two important clinical signs of SQTS are the presence of short QT intervals in normal heart structures and without other comorbidities. SQTS can be a genetic disorder that can cause repolarization abnormalities and decrease myocardial refractory period. The relationship between SCD and short QT intervals has been previously suspected,^3^ but the first clinical cases have only been reported in the last decade.^4^

Because of the limited number of cases worldwide, therefore, it is difficult to determine the real prevalence of SQTS in global population. Iribarren et al, conducted of study using a database of 6.4 million ECGs from 1.7 million persons between 1995 and 2008. It stated that the prevalence of short QT (<300 ms) was highest in blacks (5.8), followed by Caucasians (3.2), Latinos (1.8), and Asian/Pacific Islanders (1.6).^5^ Another study by Funada et al, showed that among 10,984 Japanese (male 50.2%), only three (0.03%) subjects had QTc < 300 ms.^6^

## 2 GENETIC FACTORS IN SQTS

**FIGURE 1:**
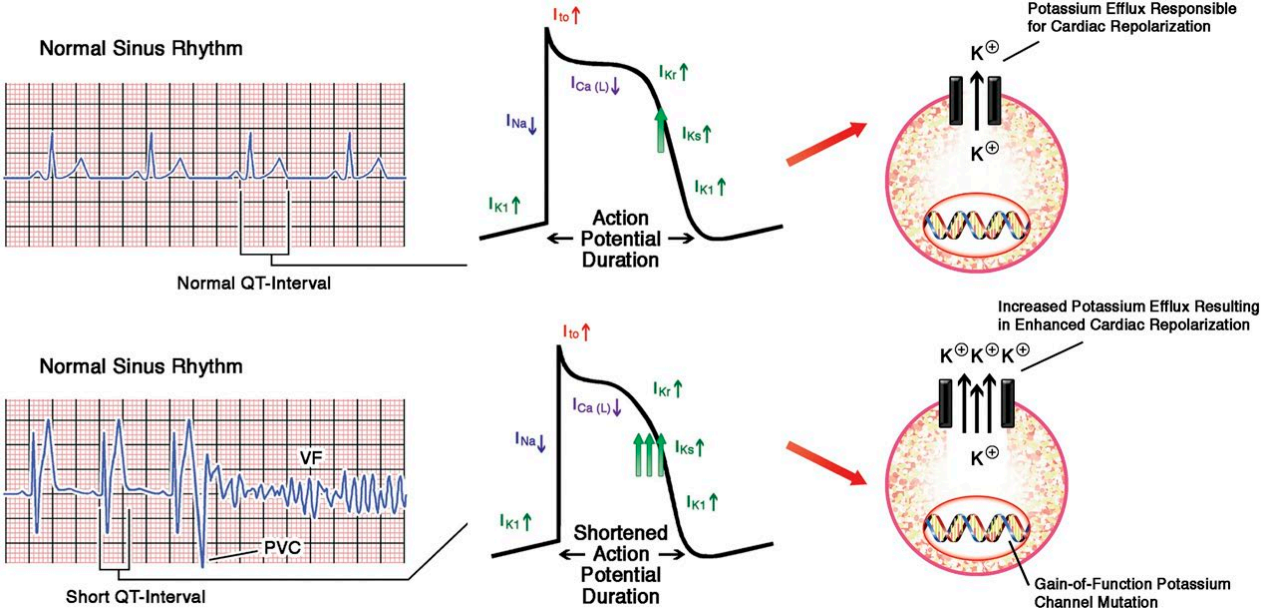
Dysfunction of the heart ion channel in SQTS ^22^

Same with other congenital arrhythmogenic abnormalities, SQTS is associated with a number of mutations that cause changes in the function of ion channels, which are responsible for regulating currents in generating cardiac action potentials. Some mutations can cause hyperfunction of delayed rectifier potassium current (I_Kr_) (Figure 1). The mutation results in increasing of transmural repolarization dispersion and shortening of the repolarization period, which explains the main features of this syndrome: short atrial‐ventricular effective refractory periods and short QT intervals, which will increase susceptibility to ventricular and atrial fibrillation (AF). SQTS is a heterogeneous disease seen both from a phenotype and genotype perspective. Six SQTS subtypes have been discovered so far according to nine mutations in six different genes that encode different cardiac ion channels (Table 1). Most SQTS are familial and the hereditary pattern is autosomal dominant. Four of the six SQTS genes are also the etiology of LQTS, but with the opposite mutation.

**TABLE 1:** SQTS classification is based on genotype

SQTS subtype	Gene	Channel function	Mechanism
SQTS 1	*KCNH2*	α‐subunit I_Kr_	Gain function
SQTS 2	*KCNQ1*	α‐subunit I_Ks_	Gain function
SQTS 3	*KCNJ1*	α‐subunit I_K1_	Gain function
SQTS 4	CACNA1C	α‐subunit I_L,Ca_	Loss function
SQTS 5	CACNB2	Β2‐subunit I_L,Ca_	Loss function
SQTS 6	CACNA2D1	Δ1‐subunit I_L,Ca_	Loss function

In 2000, Gussak et al, were the first to describe idiopathic familial short QT interval.^7^ In 2014, Brugada et al, reported SQTS 1.^8^ This variant is the most common type. It was reported that, an increase in mutations in the *KCNH2* gene resulted in a shortening of potential action in two families who were diagnosed with SQTS. The mutations cause an increased risk of SCD from a heart attack. SQTS 2 was identified by Bellocq et al, in 2004.^9^ The researchers explained alternative molecular mechanisms in patients with short QT intervals and ventricular fibrillation (VF). Increasing of KCNQ1 mutation will increase the flow of IKs. However, only a few sporadic cases of this variant have been documented. One year later, the third variant of this syndrome (SQTS 3) was described by Priori et al, in two patients.^10^ The presence of genetic changes in the *KCNJ2* gene causes a significant increase in the I_K1_ outflow, which leads to the repolarization final phase acceleration. In 2007, Antzelevitch et al, described two new variants with the same canal dysfunction: loss of mutation function in the *CACNA1C* and *CACNB2* genes encoding α1 and β2b subunits on L‐type calcium channels associated with SCD related with familial heart disease, where SQTS is combined with the phenotype of Brugada syndrome.^11^ The two mutations have been referred to, respectively, as SQTS 4 (two patients) and SQTS 5 (seven patients). Templin et al, described another mutation in the *CACNA2D1* gene that causes a decrease in the flow of Ca‐type L channels (SQTS 6). However, gene mutations are not found in all SQTS patients and the factors that related the appearance of these mutations have not been identified with certainty. This heterogeneity and the small number of cases are challenges for future research.

## 3 CLINICAL PRESENTATIONS

The clinical presentations of SQTS are quite diverse. In the case series by Giustetto C et al, the most common symptom is cardiac arrest (34%). It is also the most frequent first clinical presentation appeared (28%).^12^ Unlike LQTS, there are no special triggers for SQTS. Although SQTS usually occurs in adults, the average age is 30 years, and the age range of clinical presentation can range from a few months to the sixth decade. Events can occur while resting, during exercise, or after listening to loud sounds. Viskin et al, reported that men with idiopathic VF showed a shorter QT interval than healthy men.^13^ Other symptoms that are often documented are palpitations and syncope. In 24% of cases, syncope is the first presenting symptom.^12^ Self‐terminating VF is considered as the most likely cause of syncope. Palpitations and AF appear in >80% of cases even though the patient is still young (children and adolescents). AF is one of the main symptoms of SQTS; therefore, vigilant management is advisable in young patients with lone AF.

## 4 DIAGNOSIS

The diagnosis of SQTS is based on the patient's family history, symptoms evaluation, and 12‐lead ECG. It is important to ask patients about specific symptoms, such as palpitations and syncope, family history of syncope, sudden death, or AF at a young age. Secondary causes of short QT intervals should also be evaluated, such as hypercalcemia, hyperkalemia, hyperthermia, acidosis, and changing of autonomic tone. If no other causes are found, the patient diagnose as suspected SQTS. Schwartz's score can help classify SQTS probabilities (Table 2). From Schwartz score, we can conclude low probability of SQTS if total score ≤2, intermediate probability 3, and high probability if total score ≥4.

**TABLE 2:** Schwartz Score SQTS

Diagnostic parameters	Points
QTc, ms	
<370	1
<350	2
<330	3
J point‐T peak interval	
<120 msec	1
Clinical history*	
History of sudden cardiac arrest	2
Documented polymorphic VT or VF	2
Unexplained syncope	1
Atrial fibrillation	1
Family history*	
First‐degree or second‐degree relative with high probability of SQTS	2
First‐degree or second‐degree relative with unexplained cardiac arrest	1
Sudden infant death syndrome	1
Genotype*	
Genotype‐positive	2
Variant of unknown significance in a culprit gene	1

* A minimum of 1 point must be obtained in the electrocardiographic section in order to obtain additional points.

**FIGURE 2:**
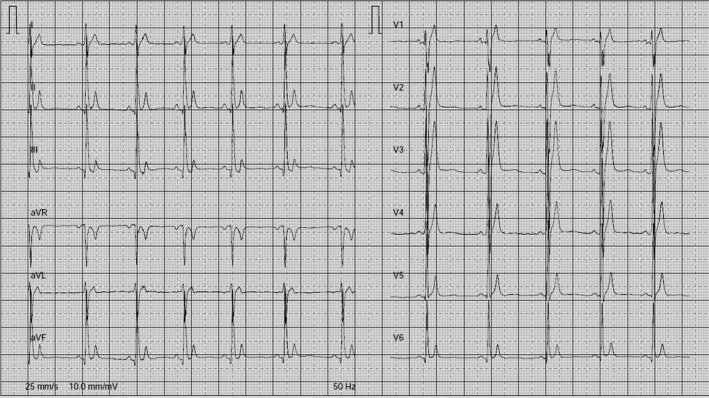
ECG taken from a 65‐y‐old patient with SQTS; QT interval 240 ms, QTc 294 ms

When evaluating ECGs in patients, three main aspects must be observed; heart rate (HR), morphology of the T wave, and duration of the QT interval (Figure 2). There is no single QTc value to distinguish the majority of SQTS cases from healthy individuals. In initial publication, patients had a short QT if the QTc value <300‐320 ms, whereas in the most recent genotype (SQTS 4 and 5), QTc <360 ms.^6^, ^7^, ^8^ In the study by Viskin et al, women with QTc <340 ms and men with QTc <330 ms can be diagnosed with SQTS even when they are asymptomatic because the values are very rare in the healthy population.^14^ In addition, QTc <360 ms (men) and <370 ms (women) can be diagnosed as SQTS when they have symptoms or family history of SCD. The European Society of Cardiology (ESC) recommends that SQTS is diagnosed if a QTc ≤340 ms (IC) is obtained.^15^ SQTS also should be considered in the presence of a QTc ≤360 ms and one or more of the following: family history of SQTS; a family history of sudden death at age 40 years; confirmed pathogenic mutation; and survival from a VT/VF episode in the absence of heart disease (IIa).

When assessing QT interval, it is also important to measure HR. Patients with SQTS usually show a constant QT value and lack of HR adaptation. There is a failure to prolong QT in bradycardia and abnormal shortening in tachycardia (QT interval pseudonormalization with fast HR). Serial ECG, Holter monitoring, and treadmill test can investigate the proper diagnosis and prevent patients from not recognizing basal heart tachycardia. In addition, this investigation can reduce incorrect diagnoses in SQTS patients with sinus bradycardia because it is known that the Bazett formula is over correct at the QT interval during bradycardia.

SQTS patients have a short or even absent ST segment, with the T wave starting immediately after the S wave. T wave is usually narrower and higher than normal subjects. Morphology of several T waves has been described to distinguish healthy subjects and SQTS. Anttonen et al, reported shorter J point‐T peak intervals and shorter T‐peak end intervals in SQTS patients.^12^ Study by Watanabe et al, observed that early repolarization was more common in SQTS patients (65%); this was associated with the appearance of arrhythmias.^16^ The duration from the peak to the end of the T wave is longer in patients with SQTS. T wave morphology can also guide a patient's genotype. SQTS has high, pointed, and symmetrical T waves. Asymmetrical T waves are sometimes obtained because of the final phase of the action potential repolarization acceleration.

The role of electrophysiological study (EPS) in SQTS is still controversial. Some researchers have shown a very short period of effective ventricular and atrial refractory, high rates of induction of ventricular and AF, and susceptibility to mechanical induction of VF. However, in the study of Giustetto et al, the sensitivity of EPSs to detect susceptibility to VF was only 50% (3/6).^12^ ESC 2015 guidelines stated that invasive EPS with programed ventricular stimulation (PVS) is not recommended for SCD risk stratification.^15^ Therefore, the benefits of examining EPS in the diagnosis and stratification of these patients are still unknown.

The genetic testing contribution is also not clearly defined. Seven genetic mutations have been identified but the correlation between genotypes and phenotypes cannot be explained with certainty. It is because the cases explained and genetically confirmed are still rare. Negative test results do not rule out SQTS because of the possibility of mutations that are not identified. Screening in one family is not only to diagnose asymptomatic congenital symptoms at an early stage but also to identify family members who do not carry certain mutations.

## 5 MANAGEMENT

Fatal arrhythmias are related with increase risk of sudden cardiac death in SQTS patients. The ESC recommends implantable cardioverter defibrillator (ICD) as SQTS (IC) therapy. ICD can be given to patients with symptoms, but doubts arise when dealing with patients without previous symptoms, especially if no previous family history is obtained. Clinical manifestations, positive EPSs, and family history or genetic testing can support ICD implantation. However, negative results do not exclude the diagnosis or the possibility of future arrhythmias.

Although ICD is an effective management, it also has a number of specific problems. Several reports indicate an increased risk of inappropriate shock due to sinus tachycardia, AF, and most importantly the presence of T waves, tall and narrow, oversensing. Study by Schimpf et al, stated that three of five patients received inappropriate shock due to T wave oversensing shortly after implantation. Even though there was no evidence of abnormalities in the predischarge test, the reason might be due to T wave signal increasing and reduction R wave amplitude.^17^ Therefore, standard programing after implantation to prevent T wave oversensing is needed.

Pharmacological therapy can be indicated as an alternative to ICD implantation in young patients (children), patients who rejected or contraindicated with ICD implantation, and to prevent symptomatic AF. Pharmacological therapy in SQTS must be given with caution because of its long‐term efficacy in preventing the incidence of serious arrhythmias only proven in SQTS 1 patients.

Quinidine is considered as the most effective pharmacological therapy in SQTS patients. This drug blocks several potassium channels (I_Kr_, I_to_, I_Ks_, I_K1_, and I_KATP_) and the flow of calcium and sodium. Quinidine has been shown to produce effective QT interval prolongation and ventricular refractory periods, ST segment lengthening and T wave duration, decreased repolarization dispersion and prevention of VF induction, and HR returning according to QT interval in SQTS 1 patients.^18^, ^19^ Wolpert et al, prove that mutations in *KCNH2* reduce the affinity of sotalol in I_Kr_ unlike quinidine.^18^ There is possibility that quinidine is superior over sotalol due to the multi‐channel effect of quinidine. ESC 2015 guidelines stated that sotalol or quinidine has class IIB recommendation for patients with SQTS diagnosis who qualify for an ICD but has a contraindication or refuse it, asymptomatic SQTS patients, and has family history of SCD (IIB).^15^ Another study stated that oral disopyramide prolongs the QT interval and ventricular effective refractory period in patients with SQTS 1. Disopyramide can be an alternative therapy besides quinidine in SQTS patients.^20^

Studies with other antiarrhythmic drugs fail to show a sufficiently good effect. Propafenone has been shown to be effective in preventing paroxysmal AF that often occurs without recurrent arrhythmias for >2 years, and without effect to QT intervals. In initial studies, flecainide seems to slightly extend the QTc interval and reduce the VF inducibility during EPSs but subsequent studies have not confirmed this effect.^21^ Ibutilide and sotalol, I_Kr_ inhibitors, have been shown to be ineffective in QT interval prolongation.

## 6 CONCLUSIONS

SQTS is a cardiac channelopathy disorder. SQTS is a life‐threatening condition that is more common in young and healthy‐looking populations. The incidental ECG findings of short QT intervals in young patients should not be underestimated, especially when associated with symptoms of arrhythmia, syncope, or the presence of paroxysmal or persistent AF documentation. Based on ESC, the diagnosis of SQTS is based on the QT interval ≤340 ms, but the diagnosis is still needed to be studied further and screening is still needed at this time. ICD is the first‐line therapy in SQTS. Until now, quinidine has been widely used as a pharmacological therapy in SQTS, especially in patients who contraindicated or rejected ICD implantation.

## CONFLICT OF INTEREST

The authors declare no conflict of interest for this article.
