# Correction to “Short QT Syndrome: The Current Evidences of Diagnosis and Management”

**DOI:** 10.1002/joa3.13043

**Published:** 2024-05-10

Dewi IP, Dharmadjati BB. Short QT syndrome: The current evidences of diagnosis and management. J Arrhythm. 2020;36(6):962–966.

In the third paragraph of the “Introduction” section, we add “Due to its rarity and potential lethality, understanding the pathogenesis and clinical implications of SQTS is crucial. This literature review aims to provide a comprehensive and detailed overview of SQTS. Additionally, this article updates prior review by Reviriego et al.,^a^ to incorporate the latest advancements and insights of SQTS.”

In the last paragraph of the “Genetic Factors in SQTS” section, we cite reference b at the end of the following sentence, as in “Templin et al, described another mutation in the CACNA2D1 gene that causes a decrease in the flow of Ca‐type L channels (SQTS 6).^b^”

We add two citations as follows.

a. Reviriego SM, Merino JL. Short QT Syndrome. ESC Council for Cardiology Practice. 2010; 9(2).

b. Templin C, Ghadri JR, Rougier JS, Baumer A, Kaplan V, Albesa M, et al. Identification of a novel loss‐of‐function calcium channel gene mutation in short QT syndrome (SQTS6). Eur Heart J. 2011;32(9):1077–88. https://doi.org/10.1093/eurheartj/ehr076

We apologize for these errors.

